# A Two-Decade Journey of Buschke-Löwenstein Tumor Without Malignant Transformation: Clinical Insights and Management

**DOI:** 10.7759/cureus.106547

**Published:** 2026-04-06

**Authors:** Chadi Bourimi, Yassine Gounni, Bakouch Mohamed, Ahmed Ibrahimi, Yassine Nouini

**Affiliations:** 1 Urology, Ibn Sina University Hospital, Rabat, MAR

**Keywords:** buschke-löwenstein tumor, giant condyloma acuminata, human papillomavirus, malignant transformation, surgical excision

## Abstract

Buschke-Löwenstein tumor (BLT), also known as giant condyloma acuminatum (GCA), represents a rare, slow-growing pseudoepitheliomatous lesion attributed to human papillomavirus (HPV) infection, characterized by local aggressiveness and potential for malignant transformation. We report a 62-year-old male with a 20-year history of progressive perineal lesions, alongside a medical background of non-muscle-invasive bladder carcinoma and chronic tobacco use. Clinical examination revealed a painless exophytic mass encompassing the perineum, scrotum, and adjacent structures; preoperative imaging and sexually transmitted infection screening yielded unremarkable findings. Histopathological analysis following wide surgical excision confirmed BLT without histological evidence of malignant transformation, with no recurrence observed at 18-month follow-up. Although an uncommon sexually transmitted entity with substantial risks of recurrence and malignant degeneration, BLT diagnosis rests upon integrated clinical and pathological correlation. Complete surgical excision remains the therapeutic cornerstone, potentially supplemented by adjunctive modalities. This case underscores the association with bladder neoplasia, likely mediated by shared risk factors including chronic inflammation and smoking, while emphasizing that early recognition and aggressive resection-even in extensive disease-can yield excellent long-term outcomes.

## Introduction

Giant condyloma acuminatum (GCA), also known as Buschke-Löwenstein tumor (BLT), is a rare, slow-growing, but locally aggressive premalignant pseudoepitheliomatous lesion caused predominantly by sexually transmitted human papillomavirus (HPV), especially types 6 and 11 [1,2]. High-risk HPV genotypes 16 and 18 may also be responsible for BLT, with oncogenesis based on viral oncoproteins E6 and E7, which inactivate p53 and Rb tumor suppressors, respectively, promoting malignant transformation [2]. Risk factors include immunosuppression, poor hygiene, smoking, chronic inflammation, diabetes, poor socioeconomic status, and multiple sexual partners [1].

It is characterized by exophytic, cauliflower-like masses arising most often in the anogenital region, with a tendency for local infiltration, recurrence, and occasional malignant transformation [3]. Although histologically benign in many cases, its clinical behavior is often destructive, making early diagnosis and complete surgical treatment essential. The disease remains challenging because of its delayed diagnosis, lack of standardized management protocols, high recurrence rates, malignant transformation potential, and impact on quality of life [1].

We report here a rare case of BLT associated with bladder tumor, an unusual coexistence that may reflect shared risk factors such as smoking and chronic inflammatory states. This case adds to the literature by providing a comprehensive review of BLT’s epidemiological, clinical, paraclinical characteristics, and therapeutic approaches, emphasizing the importance of timely diagnosis and management to reduce morbidity and recurrence risks.

## Case presentation

We present the case of a 62-year-old male, a former chronic smoker with a medical history notable for hypertension managed with amlodipine and a non-muscle-invasive bladder tumor treated by transurethral resection (TURBT) 11 months prior. The patient reported a papillomatous tumor in the perineal region, which had progressively enlarged over 20 years, although he remained generally in stable health.

**Figure 1:**
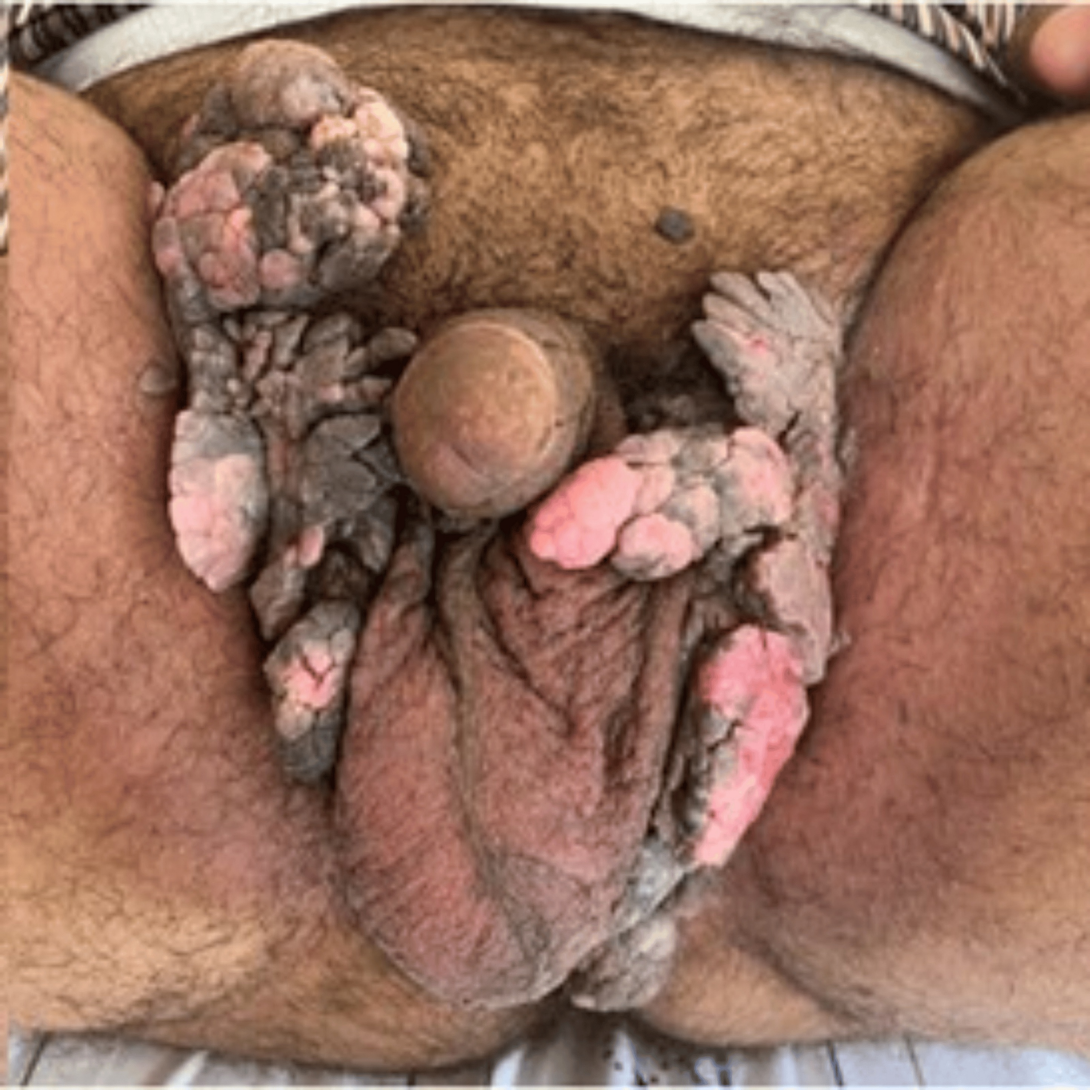
Cauliflower-like papillomatous lesions of the perineum, scrotum, and base of the penis, with inguinal extension.

Clinical examination revealed a large, multifocal exophytic lesion with estimated cumulative dimensions of approximately 14 × 12 × 8 cm (precise measurement difficult due to confluent lesions spanning the perineal region), exhibiting a classic papillomatous cauliflower-like appearance. The lesion showed an infiltrative growth pattern and emitted a fetid odor but was painless. It predominantly involved the perineum, extending to the penile base, scrotum, inguinal area, and perianal region. While intermittent contact bleeding occurred, no ulcerations, fistulas, or collections were observed (Figure 1). Regional lymph nodes were clinically unremarkable.

Comprehensive screening for sexually transmitted infections yielded negative results. Preoperative computed tomography (CT) of the pelvis was performed and did not demonstrate deep invasion of the underlying soft tissues or involvement of pelvic organs; however, pelvic magnetic resonance imaging (MRI) was not obtained in this case.

**Figure 2:**
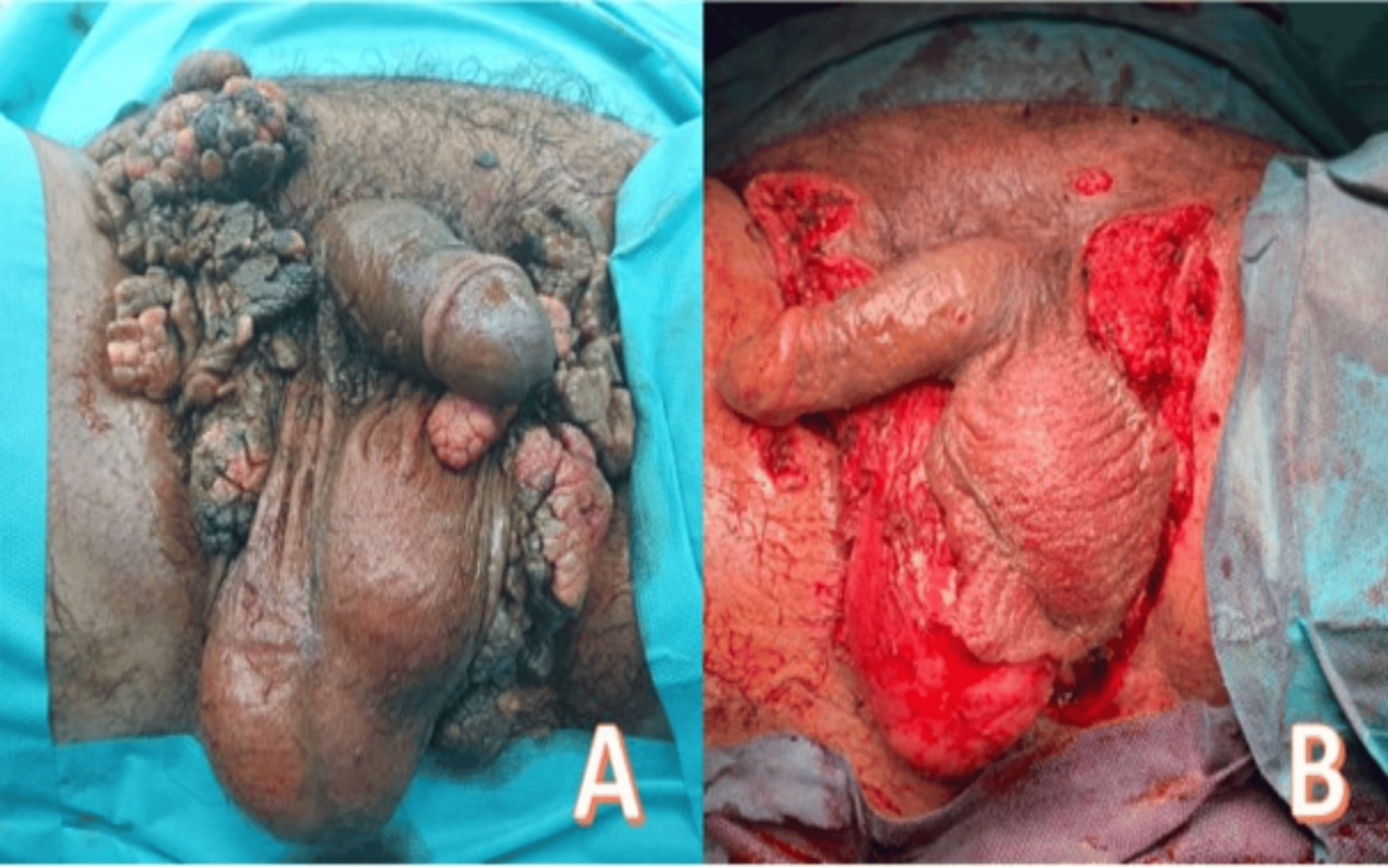
A: Patient set-up in the operating room in the lithotomy position. B: Postoperative aspect after removal of lesions by conventional surgery.

Wide surgical excision of the tumor with clear margins was performed using conventional electrocautery dissection (Figure 2). The resulting defect was partially closed with interrupted absorbable sutures in tension-free areas, with the remaining surfaces managed by secondary intention healing, leading to complete epithelialization without complications. The postoperative recovery was uneventful.

**Figure 3:**
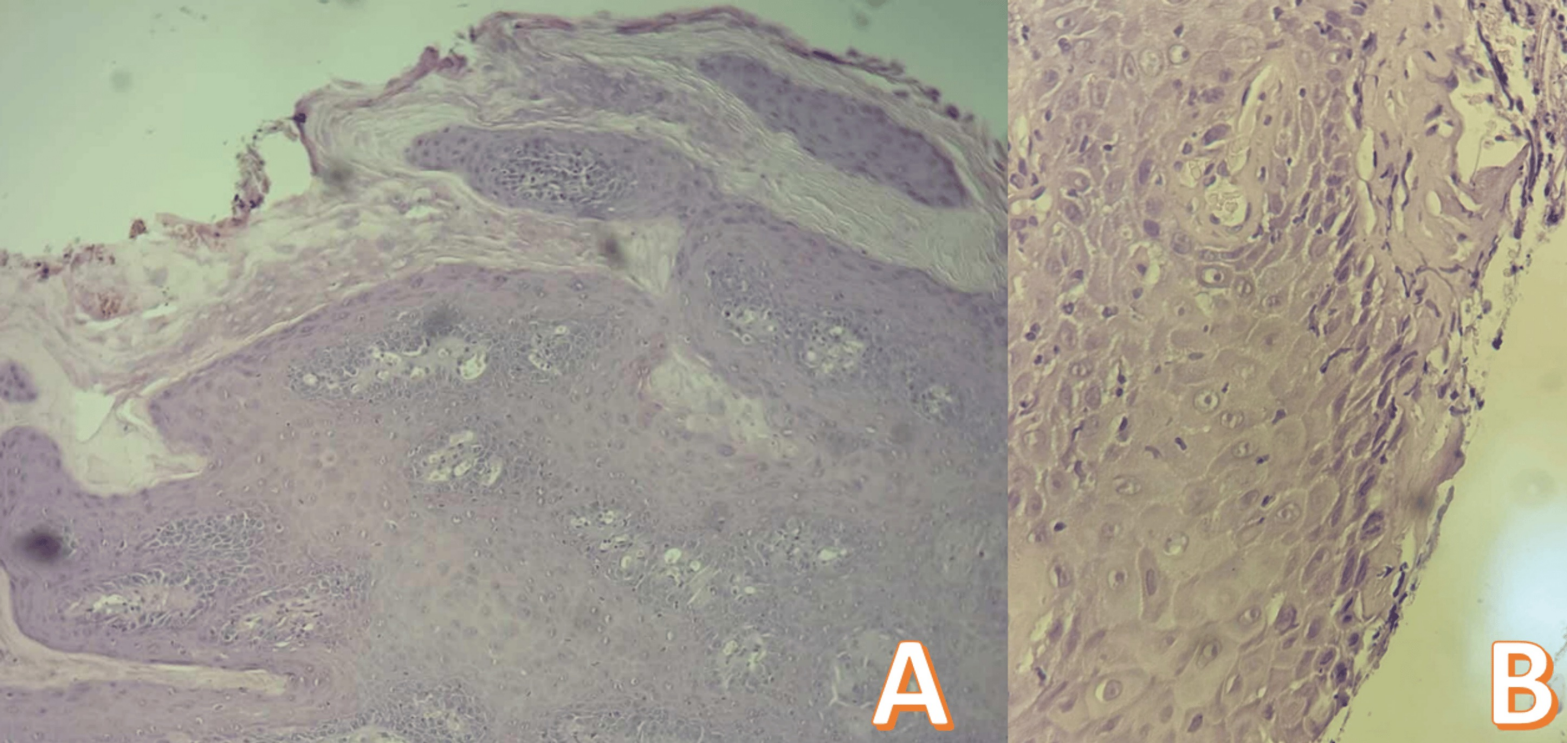
Histopathological findings (hematoxylin & eosin staining). A (Low magnification): Low-power view revealing exophytic squamous proliferation with marked papillomatosis, acanthosis, hyperkeratosis, and prominent fibrovascular cores extending into the dermis. B (High magnification): High-power view showing koilocytes with perinuclear halos, parakeratosis, and minimal cellular atypia without evidence of frank malignancy.

Histopathological examination of the entire excised specimen (standard hematoxylin-eosin staining) confirmed the diagnosis of BLT with negative surgical margins. It revealed an exophytic verrucous squamous proliferation with marked papillomatosis, acanthosis, hyperkeratosis, and fibrovascular cores extending into the dermis. Well-differentiated hyperplastic epithelium showed koilocytes with perinuclear halos, abundant basaloid mitoses, and an intact basement membrane, consistent with HPV-driven benign yet locally aggressive changes (Figure 3). No additional immunohistochemistry (e.g., Ki-67, p16) was performed, as H&E showed no cytological atypia, dysplasia, or stromal invasion diagnostic of malignant transformation. Specific HPV typing was not performed. Importantly, no histological evidence of malignancy was identified.

**Figure 4:**
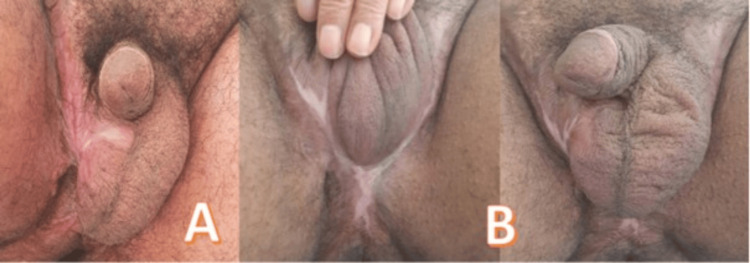
Evolution aspects after a follow-up of 12 months (A) and 18 months (B).

Subsequent to the therapeutic intervention, follow-up assessments were conducted, revealing no evidence of tumor recurrence at the 18-month mark, thus underscoring the effectiveness of the therapeutic approach employed (Figure 4).

## Discussion

BLT is a rare dermatological condition first documented in 1896 and subsequently recognized as distinct from verrucous carcinoma [4]. It is characterized by cauliflower-like exophytic lesions that typically infiltrate adjacent tissues, with recurrence rates reaching 67% and a malignant transformation risk of 30-56% [3,5]. These attributes substantially contribute to morbidity, diminished quality of life, and mortality rates of 20-30% [3].

BLT exhibits an estimated population prevalence of 0.1%, predominantly affecting middle-aged males (mean age 44 years) at a male-to-female ratio of 2.7:1, although pediatric cases underscore its diverse clinical manifestations [4]. While sexual contact constitutes the primary transmission route, documented instances of autoinoculation and heteroinoculation indicate multiple pathways of transmission [4,6].

HPV types 6 and 11 are implicated in over 90% of BLT cases [4]. Associated risk factors encompass immunosuppression, poor hygiene, diabetes, alcoholism, multiple sexual partners, chronic genital inflammation, anaerobic infections, and smoking [7]. Despite this established association, the efficacy of HPV vaccination in preventing BLT remains uncertain, owing to the lesion’s rarity [4].

The coexistence of bladder tumor and BLT, as observed in this case, represents a rare phenomenon that may occur more frequently among immunocompromised patients with chronic inflammation. Shared risk factors, particularly smoking, may contribute to the concurrent development of bladder cancer and HPV-related lesions such as BLT. Although a direct causative link has not been established, underlying factors that promote both oncogenesis and viral infection likely play a contributory role. This notable overlap, evident in our patient, underscores the importance of comprehensive risk assessment in affected individuals.

Clinically, BLT typically manifests as a verrucous, exophytic mass on the external genitalia, with anorectal involvement reported in 10-17% of cases [7]. Associated symptoms may include dysuria, constipation, abdominal distension, and hemorrhoids [4]. Extra-genital localizations, such as the axillary region, are rare but pose unique anatomical challenges for surgical management [1,4]. The present case exemplifies classic BLT features-impressive cauliflower-like growth with local invasion yet low metastatic potential-consistent with the published literature [5]. The patient's 20-year delay in seeking treatment stemmed from the absence of pain or systemic symptoms, compounded by social stigma associated with genital conditions, thereby underscoring significant barriers to timely diagnosis.

Advanced imaging modalities, including CT and MRI, play a crucial role in delineating local and systemic involvement. In this case, preoperative pelvic CT revealed no evidence of deep soft tissue invasion or pelvic organ involvement; however, an MRI was not performed.

Standard hematoxylin and eosin (H&E) staining provides a reliable diagnosis of BLT through characteristic findings of koilocytes, papillomatosis, and acanthosis, while distinguishing it from verrucous carcinoma by the absence of cytological atypia and stromal invasion [2]. In equivocal cases, immunohistochemical (IHC) markers such as Ki-67 (proliferation index) and p16 (high-risk HPV marker) help evaluate tumor aggressiveness and transformation risk; however, classic H&E features without atypia - as observed here - preclude the need for IHC [3].

The malignancy of BLT may imply the presence of histological evidence of malignancy and/or deep infiltration of adjacent tissues. Histological evidence of malignancy has been found in 50% of patients exhibiting infiltrative disease [8,9]. A limitation of this case is the absence of immunohistochemical markers and advanced imaging such as MRI, which could have offered a more comprehensive evaluation of tissue invasion.

Treatment strategies for BLT are diverse and individualized according to lesion size, location, and previous interventions. Wide surgical excision with negative margins remains the gold standard, whether employed as monotherapy or in combination with adjuvant or neoadjuvant therapies, as clear margins substantially reduce recurrence rates [10,11]. Although alternative techniques-including electrocoagulation, radiofrequency ablation, and carbon dioxide laser ablation-have been proposed, their relative efficacy remains undetermined due to insufficient comparative studies [3,4]. Complete lesion excision may necessitate reconstructive procedures [5]. Ultimately, optimal treatment selection hinges on individual patient characteristics in the absence of standardized therapeutic guidelines.

Adjunctive therapies encompass chemotherapy and radiotherapy, with protocols incorporating 5-fluorouracil and mitomycin receiving priority [4]. Neoadjuvant approaches are particularly suited to unresectable or malignant cases to reduce tumor volume prior to surgery [4].

Topical 5% imiquimod cream, an immune response modifier, demonstrates efficacy in multimodal regimens, although it is contraindicated on mucosal surfaces due to inflammatory risks [4,12]. While podophyllin's efficacy as monotherapy remains inconclusive, combination protocols incorporating podophyllin, imiquimod, and cryotherapy have been employed for extra-genital BLT when surgical intervention is limited by functional constraints [13].

The complete surgical excision performed in this case aligns with contemporary preferred strategies and correlates with favorable outcomes reported across multiple reports, including lesion resolution without recurrence in many patients [14,15]. The patient's uneventful postoperative recovery underscores the value of individualized treatment planning, particularly in cases characterized by prolonged disease duration. These findings reinforce current best practices while demonstrating the potential for curative intervention in carefully selected patients. Nevertheless, the lack of standardized treatment protocols highlights the pressing need for additional research to develop evidence-based guidelines.

In comparison to other reported cases, this presentation is distinguished by its exceptionally prolonged lesion duration (20 years) without malignant transformation, alongside the uncommon coexistence of BLT with bladder carcinoma, suggesting shared risk factors that remain infrequently addressed in the literature. The patient's 20-year delay in pursuing surgical intervention exemplifies the diagnostic challenges inherent to BLT. Contributing factors included the absence of pain, minimal disruption to daily activities, and pervasive social stigma associated with genital pathology, all of which facilitated lesion progression. These elements, consistently documented across studies, emphasize the critical need for heightened awareness among at-risk populations to promote earlier detection and optimal therapeutic outcomes.

Despite therapeutic advances, the prognosis of BLT remains challenging owing to elevated recurrence rates and associated complications. Recurrence occurs in approximately 67% of cases, including up to 50% of those undergoing radical surgery [4,14]. This underscores the necessity for long-term surveillance to monitor for recurrence and manage potential sequelae, which may include fistulas, abscesses, urethral stenosis, urinary tract infections, anal incontinence, and anal stenosis [3]. Follow-up should rely on repeated clinical assessment and, when needed, imaging, especially during the first few years when relapse is most likely.

Mortality related to BLT is estimated at 21%, predominantly driven by comorbid conditions, with higher rates (33%) observed among patients lacking malignant transformation compared to those exhibiting it (13%) [4,5].

The profound impact of BLT on the quality of life, stemming from persistent symptoms, recurrent disease, surgical interventions, and chemotherapy, underscores the imperative for comprehensive management that addresses both physical and psychological aspects.

## Conclusions

BLT represents an uncommon yet clinically significant entity characterized by large, recurrent verrucous tumors predominantly associated with HPV infection. Although standardized treatment protocols remain elusive, complete surgical excision with clear margins, as successfully achieved in this case, constitutes the preferred therapeutic approach and may be augmented by adjuvant therapies such as radiotherapy or chemotherapy in select circumstances. Optimal management necessitates a multidisciplinary strategy to address the multifaceted clinical challenges posed by BLT.
